# Bartter and Gitelman syndromes: Questions of class

**DOI:** 10.1007/s00467-019-04371-y

**Published:** 2019-10-29

**Authors:** Martine T. P. Besouw, Robert Kleta, Detlef Bockenhauer

**Affiliations:** 1 Department of Pediatric Nephrology, University of Groningen, University Medical Center Groningen, Groningen, The Netherlands; 2 Renal Unit, Great Ormond Street Hospital for Children NHS Foundation Trust, London, UK; 3 Department of Renal Medicine, University College London, London, UK

**Keywords:** Bartter syndrome, Gitelman syndrome, EAST syndrome, Tubulopathy, Metabolic alkalosis, Hypokalaemia

## Abstract

Bartter and Gitelman syndromes are rare inherited tubulopathies characterized by hypokalaemic, hypochloraemic metabolic alkalosis. They are caused by mutations in at least 7 genes involved in the reabsorption of sodium in the thick ascending limb (TAL) of the loop of Henle and/or the distal convoluted tubule (DCT). Different subtypes can be distinguished and various classifications have been proposed based on clinical symptoms and/or the underlying genetic cause. Yet, the clinical phenotype can show remarkable variability, leading to potential divergences between classifications. These problems mostly relate to uncertainties over the role of the basolateral chloride exit channel CLCNKB, expressed in both TAL and DCT and to what degree the closely related paralogue CLCNKA can compensate for the loss of CLCNKB function. Here, we review what is known about the physiology of the transport proteins involved in these disorders. We also review the various proposed classifications and explain why a gene-based classification constitutes a pragmatic solution.

## Introduction

### Physiology and pathophysiology

Small solutes like sodium and potassium are freely filtered by the glomerulus and will subsequently be reabsorbed in different parts of the nephron. Approximately 99% of the total amount of filtered sodium will be reabsorbed: around 70–80% in the proximal tubule (PT), 10–20% in the thick ascending limb of the loop of Henle (TAL), another 5–10% in the distal convoluted tubule (DCT), and around 2–5% in the collecting duct (CD). The percentages of potassium being reabsorbed in the PT and TAL are more or less comparable to those of sodium. However, in the more distal DCT and the CD, also known as the aldosterone-sensitive part of the nephron, potassium can be actively secreted. Inherited or acquired factors (the latter including drugs) that disrupt the function of specific transporters in these nephron segments can cause a range of symptoms, the most common being polyuria and polydipsia, electrolyte abnormalities, and acid-base disturbances [1–4].

### Salt reabsorption in the TAL

**Fig. 1.**
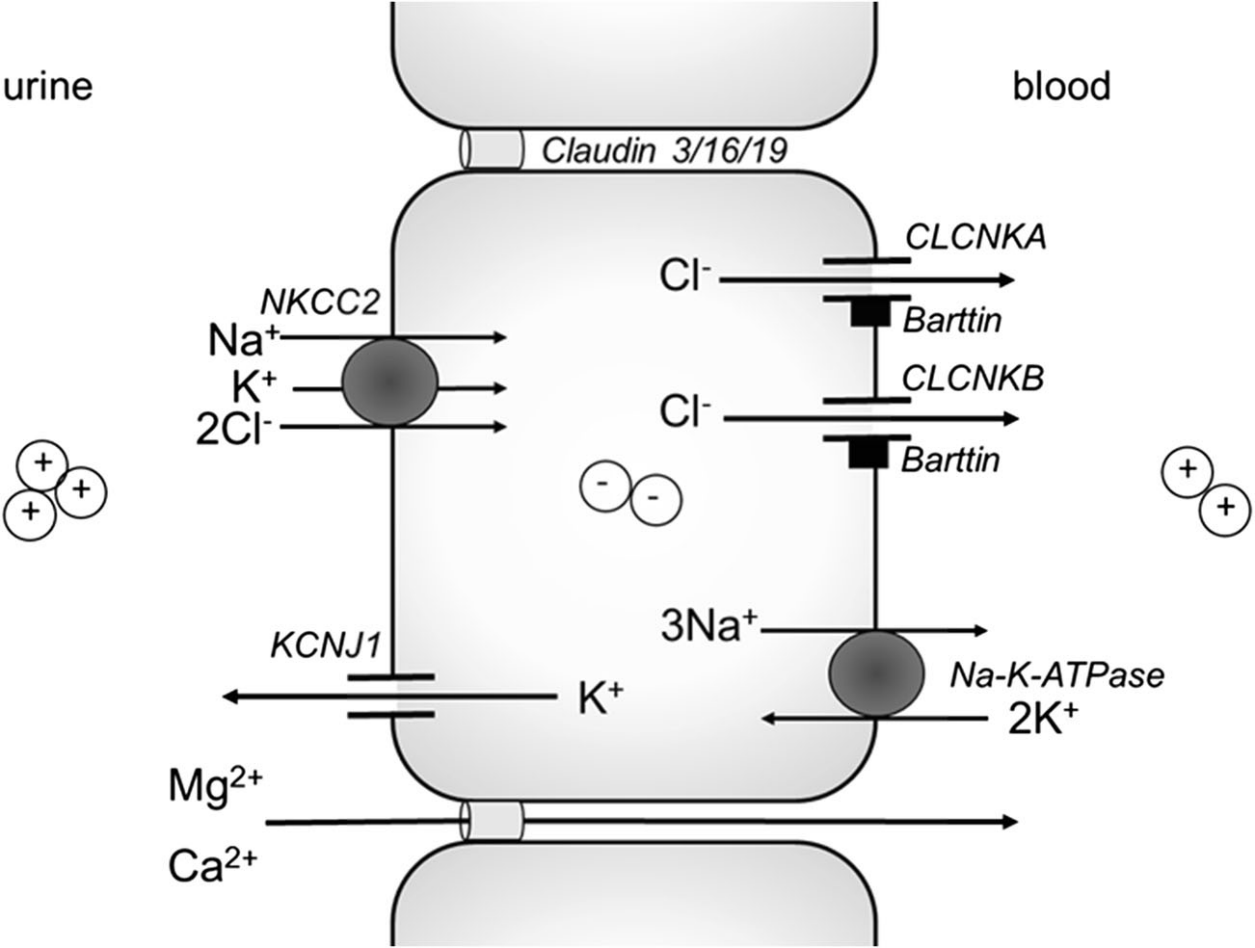
Electrolyte transport in the TAL. The NKCC2 transporter imports one sodium, one potassium, and two chloride ions from the lumen into the cell. This transporter can be blocked by loop diuretics, inherited dysfunction causes Bartter syndrome type 1. Mutations in the gene encoding MAGE-D2 result in a transient decreased expression of NKCC2, thus causing Bartter syndrome type 5. Chloride leaves the cell via the basolateral chloride transporters CLCNKB and, presumably, CLCNKA, both of which need the Barttin subunit in order to function. Mutations in CLCNKB cause Bartter syndrome type 3 and in Barttin cause Bartter syndrome type 4a; combined dysfunction of the CLCNKA and CLCNKB channels causes Bartter syndrome type 4b. Sodium leaves the cell via the basolateral Na-K-ATPase that actively exports three sodium ions and imports two potassium ions. Potassium subsequently leaves the cell via the luminal KCNJ1 channel, mutations in which cause Bartter syndrome type 2. The luminal potassium concentration is the main driving force for the paracellular uptake of calcium and magnesium, which is facilitated by a lumen-positive transepithelial potential. Therefore, hypercalciuria with nephrocalcinosis and hypermagnesuria is typically seen in Bartter syndromes type 1 (NKCC2), type 2 (KCNJ1), and type 5 (MAGE-D2), while it can be variable in Bartter syndromes type 3 (CLCNKB), type 4a (Barttin), and type 4b (combined CLCNKA and CLCNKB)

In the TAL, sodium is reabsorbed from the tubular lumen into the cell together with potassium and two chloride molecules by the luminal sodium-potassium-2 chloride cotransporter (NKCC2), which can be blocked by loop diuretics (Fig. 1). Next, chloride leaves the cell via basolateral chloride channels, mainly CLCNKB and, presumably to a lesser degree, also through CLCNKA. The uncertainty about the exact nature of the chloride exit pathway stems mainly from the fact that these 2 chloride channels are highly homologous. They are encoded by 2 adjacent genes on chromosome 1, the result of an ancient gene duplication [5]. Thus, antibodies that can distinguish between these channels have not yet been identified, making exact localisation difficult. Using mostly a technique called in situ hybridization, which assesses mRNA expression, some differences in localization have been reported, showing that CLCNKA (or its orthologues in rodents) is mainly expressed in the loop of Henle, including the thin descending limb, whereas CLCNKB is predominantly expressed in the thick ascending limb, as well as the DCT, but also in the collecting duct [5]. Clues for the exact roles of these channels in man are mainly inferred from clinical observations. Loss of function of CLCNKB causes Bartter syndrome type 3 (BS3), which clearly establishes CLCNKB as the key exit pathway for chloride in the TAL. In contrast, isolated loss of function of CLCNKA has not yet been associated with a clinical phenotype in man, despite actively looking for mutations in the gene encoding this transporter in unexplained cases of Bartter syndrome. If CLCNKA is knocked-out in mice, there is impairment of urinary concentration, but no apparent salt wasting [6]. Yet, the combined loss of function of both channels causes a more severe type of Bartter syndrome, so-called Bartter syndrome type 4b (BS4b), suggesting that CLCNKA does have a complementary role in mediating chloride exit [7]. Both CLCNKA and CLCNKB channels depend on the presence of a subunit called Barttin to enable chloride transport. Loss of function of this subunit causes Bartter syndrome type 4a (BS4a), which is clinically similar to BS4b [8]. Sodium is then transported out of the cell by the basolateral Na-K-ATPase. This transporter is present in the cell membrane of virtually all cells in our body in order to maintain the electrochemical gradients for sodium and potassium crucial for cell function. It actively transports three sodium molecules out and two potassium molecules into the cell. Lastly, potassium recycles back into the tubular lumen via the potassium channel KCNJ1 (also called ROMK). This recycling establishes the lumen-positive transepithelial potential that is needed for the paracellular uptake of cations, including calcium and magnesium via claudins (Fig. 1) [2–4]. It is important to note that the macula densa, the site of tubuloglomerular feedback (TGF) is part of the TAL. Thus, impaired salt reabsorption in TAL leads to a “short-circuiting” of TGF with increased renin production, which is irrespective of volume status [9–11].

#### The clinical consequences of impaired TAL salt transport: the “loop” phenotype

Impaired salt reabsorption in the TAL leads to specific clinical consequences that can be explained by the underlying molecular physiology: the loss of salt, as well as the “short-circuited” TGF lead to highly elevated renin and aldosterone levels causing hypokalaemic, hypochloraemic metabolic alkalosis. In addition, the loss of the lumen-positive transepithelial voltage gradient leads to impaired paracellular cation uptake, mostly manifesting as hypercalciuria. Lastly, as the TAL is critical for urinary concentration by dilution of tubular fluid and generation of the interstitial concentration gradient, patients have a urinary concentrating defect, typically isosthenuria, although some have hyposthenuria [12]. The pharmacologic equivalent to the “loop” phenotype are obviously loop diuretics, the use of which results in the same blood and urine electrolyte abnormalities [13].

### Salt transport in the DCT

**Fig. 2.**
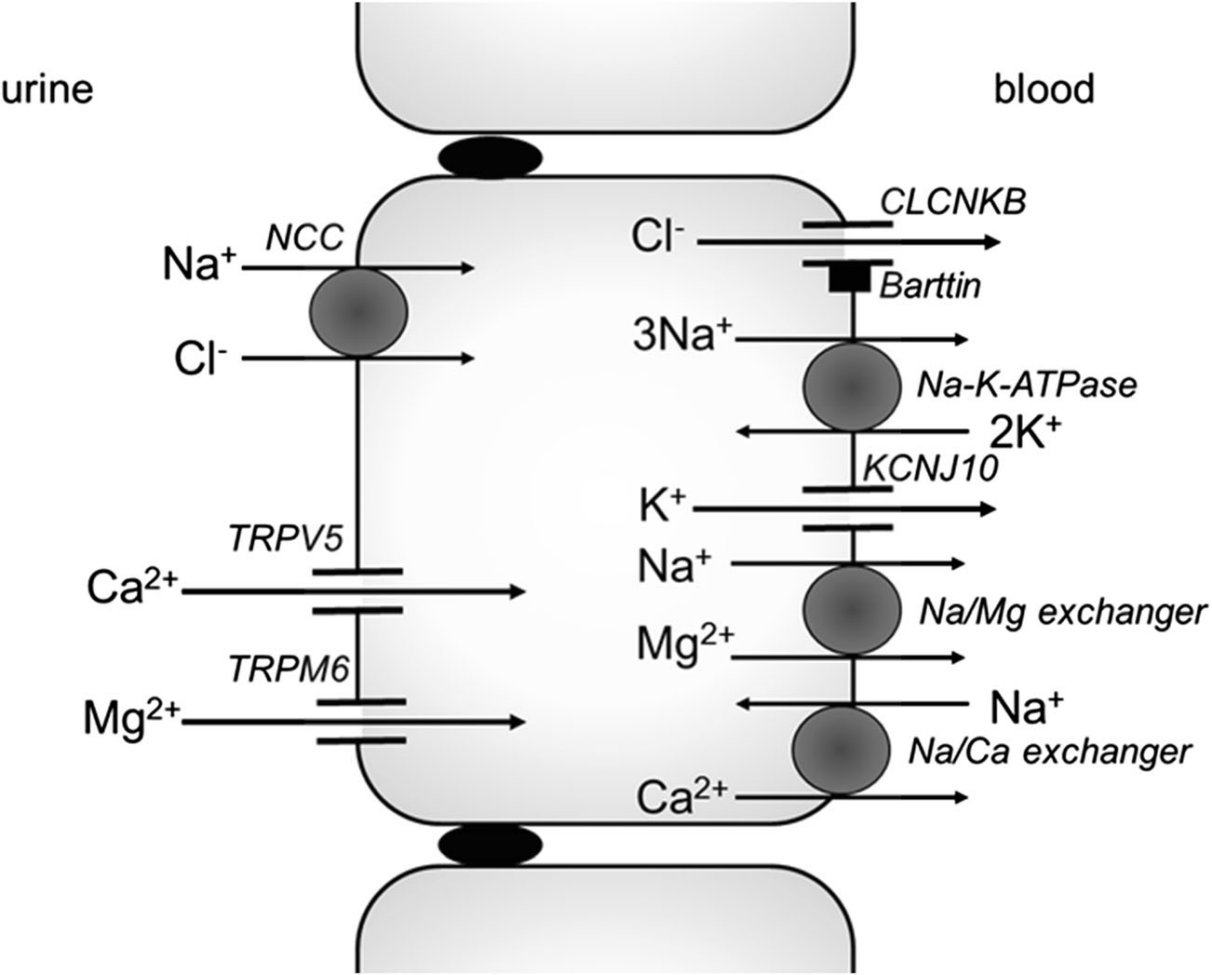
Electrolyte transport in the DCT. The NCC transporter imports one sodium and one chloride ion from the lumen into the cell. This transporter can be blocked by thiazide diuretics. Mutations in the gene encoding NCC cause Gitelman syndrome. Chloride leaves the cell via the basolateral CLCNKB channel, which needs the Barttin subunit in order to function. Both proteins are expressed in both the TAL and DCT, and their decreased function can cause Bartter syndrome (type 3 and type 4a, respectively). Sodium can exit the cell via the basolateral Na-K-ATPase that actively exports three sodium ions while importing two potassium ions. Potassium is immediately recycled and pumped out of the cell by the basolateral potassium channel KCNJ10, mutations in which cause EAST syndrome. Calcium and magnesium are imported by TRPV5 and TRPM6, respectively.

In the DCT, sodium and chloride are transported into the cell by the luminal sodium-chloride cotransporter (NCC), which can be blocked by thiazide diuretics (Fig. 2). Chloride subsequently exits the cell by the basolateral CLCNKB with its Barttin subunit. Again, sodium is exported and potassium is imported by the basolateral Na-K-ATPase. The activity of this transporter in the DCT depends on the activity of the basolateral potassium channel KCNJ10 (also called Kir4.1), which enables the recycling of potassium across the basolateral membrane, thus ensuring a steady supply for the Na-K-ATPase. The importance of this basolateral recycling is highlighted by loss-of-function mutations in this gene leading to EAST (also called SeSAME) syndrome, which includes a Gitelman-like tubulopathy [14, 15]. In this part of the nephron, calcium and magnesium are reabsorbed via the transcellular route. Calcium enters the cell via the luminal transient receptor potential cation channel subfamily V member 5 (TRPV5). It leaves the cell via the basolateral Na/Ca exchanger, which removes calcium from the cell in exchange for sodium. Magnesium is imported by the luminal transient receptor potential cation channel subfamily M member 6 (TRPM6). The basolateral exit pathways are thought to include a basolateral Na/Mg exchanger [2–4, 16–18].

#### The clinical equivalent: the “DCT” phenotype

Just as with loop dysfunction, impaired salt reabsorption in the DCT causes characteristic electrolyte abnormalities. The impaired salt reabsorption and consequent volume loss leads to increased renin and aldosterone levels, resulting in the hypokalaemic, hypochloraemic metabolic alkalosis, common to Bartter, Gitelman, and EAST syndromes. Yet, in contrast to loop dysfunction, TGF is intact. Similarly, paracellular calcium reabsorption in the TAL is unaffected and patients typically have hypocalciuria, presumably due to a compensatory increase in salt, and thus calcium reabsorption in the proximal tubule [19].

### Bartter syndromes

Bartter syndrome (BS) was first described in 1962 by Frederic Bartter [20]. Patients present with polyuria (which, depending on the subtype, can manifest antenatally with polyhydramnios and preterm birth), hypokalaemic, hypochloraemic metabolic alkalosis and normal blood pressure in the context of elevated renin and aldosterone levels. On a genetic basis, 5 different subtypes are currently distinguished (Table 1) [3, 4, 21].

**Table 1:** Bartter, Gitelman, and EAST syndrome classification according to gene defect and clinical manifestation

Disorder	OMIM	Inheritance	Gene	Protein	First presentation	Specific clinical findings^#^
Bartter syndrome type 1	601678	AR	*SLC12A1*	NKCC2	Antenatal	Nephrocalcinosis
Bartter syndrome type 2	241200	AR	*KCNJ1*	KCNJ1	Antenatal	Postnatal transient hyperkalaemia, nephrocalcinosis
Bartter syndrome type 3	607364	AR	*CLCNKB*	ClC-Kb	Variable	Variable
Bartter syndrome type 4a	602522	AR	*BSND*	Barttin	Antenatal	Sensorineural deafness, severe polyhydramnios
Bartter syndrome type 4b	613090	AR	*CLCNKA* and CLCNKB*	ClC-Ka and ClC-Kb	Antenatal	Sensorineural deafness, severe polyhydramnios
Bartter syndrome type 5	300971	XLR	*MAGED2*	MAGE-D2	Antenatal	Transient Bartter syndrome, nephrocalcionsis
Gitelman syndrome	263800	AR	*SLC12A3*	NCCT	Childhood	Hypomagnesemia, hypocalciuria
EAST syndrome	612780	AR	*KCNJ10*	Kir4.1	Infancy	Epilepsy, ataxia, sensorineural deafness

^#^Besides hypokalaemic, hypochloraemic metabolic alkalosis

*Simultaneous mutations. AR, autosomal recessive; AD, autosomal dominant; XLR, X-linked recessive; NKCC2, Na-K-2Cl cotransporter; CLCNKA, kidney chloride channel a; CLCNKB, kidney chloride channel b; MAGE-D2, melanoma-associated antigen D2; NCC, Na-Cl cotransporter; Na-K-2Cl cotransporter, Na-K-2Cl cotransporter

Bartter syndrome type 1 (BS1) is caused by mutations in the gene encoding the luminal NKCC2 transporter, causing polyhydramnios and premature birth. Patients have hypercalciuria and nephrocalcinosis.

Bartter syndrome type 2 (BS2) is caused by mutations disrupting the function of the luminal KCNJ1 channel, again resulting in polyhydramnios and prematurity. Patients with BS2 also show hypercalciuria and nephrocalcinosis. In addition, children with this type of BS typically have transient hyperkalaemia in the neonatal period, as KCNJ1 also constitutes a major pathway for potassium secretion in the collecting duct. After the immediate neonatal period, other potassium channels, most notably the so-called BK channels can compensate for the loss of KCNJ1 in the CD; yet, patients with BS2 typically have the highest plasma potassium levels of all BS subforms [21, 22]. Because of the common presentation with polyhydramnios and prematurity, BS1 and BS2 are often classified as “antenatal BS” [13].

Bartter syndrome type 3 (BS3) is sometimes also referred to as “classical BS,” as patients with this subform typically present postnatally and are thus closer to the original description by Bartter. BS3 is caused by a defective function of the basolateral chloride channel CLCNKB, which is expressed both in the TAL and the DCT. Affected children are usually born at term and present in childhood with the typical electrolyte abnormalities. However, more severe phenotypes including antenatal presentation with polyhydramnios have been reported in a subset of patients. They can show hypercalciuria and nephrocalcinosis, as well as hypomagnesemia.

Bartter syndrome type 4a (BS4a) is caused by disrupting mutations in the gene encoding Barttin, a subunit of the basolateral chloride channels CLCNKA and CLCNKB. Type 4b (BS4b) is caused by simultaneous mutations in genes encoding both CLCNKA and CLCNKB, giving a phenotype similar to BS4a. Children with BS4 (a or b) typically present with polyhydramnios and premature birth and can therefore also be classified as “antenatal BS.” Patients can have hypercalciuria and nephrocalcinosis. Moreover, hypomagnesemia can be found. Both these subtypes of BS are accompanied by sensorineural deafness, since both chloride channels and their Barttin subunit are expressed in the inner ear where the efflux of chloride through these channels is needed to enable depolarization of hair cells. Deafness only occurs if function of both types of chloride channels is impaired, thus severely hampering chloride transport. This is the case in BS4 but not in BS3 where chloride transport via CLCNKA is still intact [23].

All the previous described subtypes of BS are inherited in an autosomal recessive manner. Recently, a transient but severe antenatal form of X-linked BS was described, caused by mutations in the gene encoding the protein melanoma-associated antigen D2 (MAGE-D2). Patients present antenatally with severe polyhydramnios causing premature birth. Again, there can be hypercalciuria, hypermagnesuria and nephrocalcinosis. This entity is now considered the fifth subtype of Bartter syndrome (BS5) [24].

Finally, autosomal dominant or familial hypocalcaemia can be associated with hypokalaemic, hypochloraemic metabolic alkalosis. Before the discovery of *MAGED2* mutations, this condition was sometimes referred to as Bartter syndrome type 5, but is nowadays considered to be a Bartter-like subform of familial hypocalcaemia. It is caused by an activating mutation in the gene encoding the basolateral calcium-sensing receptor (CaSR). Once activated, CaSR reduces the activity of KCNJ1, NKCC2, and the Na-K-ATPase, thereby causing a phenotype that can mimic BS. For this reason, this disorder has sometimes been called BS type 5, but in the database Online Mendelian Inheritance in Man (OMIM; www.omim.org), this classification is now reserved to patients with mutations in *MAGED2*, as discussed above. This is presumably to avoid assignation of two different names (autosomal dominant hypocalcaemia and BS5) to the same disease.

Patients with autosomal dominant hypocalcaemia can present at any age, but usually become symptomatic during adolescence or in adulthood. In contrast to patients with BS, who in general are normocalcaemic, patients with autosomal dominant hypocalcaemia usually present with symptomatic hypocalcaemia and hypercalciuria causing nephrocalcinosis. Moreover, it has been shown that CaSR activation decreases the activity of the basolateral inwardly rectifying potassium channel KCNJ10, which ensures the recycling of potassium over the basolateral membrane in order to maintain Na-K-ATPase activity. The effect of CaSR activation on the DCT can explain the hypomagnesemia that is often seen in patients with familial hypocalcaemia [25, 26].

### Gitelman syndrome

Gitelman syndrome (GS) was first described in 1966 by Hillel Gitelman [27] (Table 1). It was initially thought to be a subtype of BS, given the similar clinical picture with hypokalaemic, hypochloraemic metabolic alkalosis, but was established as a separate entity by molecular genetics. It is caused by mutations in the gene *SLC12A3,* encoding NCC and is inherited in an autosomal recessive manner. As this transporter is only expressed in DCT, GS is exclusively a disorder of DCT. Patients typically present in late childhood or adulthood with hypokalaemic, hypochloraemic metabolic alkalosis and normal blood pressure despite hyperaldosteronism, which can also be found in BS. Key features at presentation in GS, however, are hypocalciuria (as opposed to the hypercalciuria in BS) with hypermagnesuria. In contrast to BS, overt hypomagnesaemia is typical for patients with GS. Chronic hypomagnesaemia is thought to cause the development of chondrocalcinosis that can be a complication in adult patients with GS. There can be some degree of polyuria and polydipsia, but this is generally less profound compared to patients with BS and, at least in childhood, urinary concentrating ability appears intact [21, 28].

Children with BS and GS have a reduced reabsorption of sodium in the TAL and DCT, respectively. This results in an increased tubular flow rate and an increased delivery of sodium to the more distal nephron. In combination with activation of the renin-angiotensin-aldosterone system (RAAS) caused by chronic volume depletion and/or TGF dysfunction, this will stimulate the reabsorption of sodium in the aldosterone-sensitive part of the nephron with a concurrent increase in the excretion of potassium and hydrogen ions, thus causing hypokalaemic alkalosis [2]. Despite this pathological hyperaldosteronism, patients with BS and GS are generally normo- or even hypotensive. This can be explained by the underlying tubulopathy, causing renal salt wasting. Moreover, patients with BS and GS are found to have increased levels of angiotensin-converting enzyme 2 (ACE2), which results to increased conversion of angiotensinogen into the vasodilatory peptide angiotensin 1–7. This opposes the effect of ACE, which converts angiotensinogen into the vasoconstrictive peptide angiotensin II [29]. Nevertheless, although hypertension in childhood is rare, some adult patients with GS develop hypertension. In one report, around 40% of adult patients with GS suffer from high blood pressure [30]. Also, mutations can be found in genes causing BS and GS in patients presenting with unexplained hypokalaemia and hypertension [31]. The exact etiology of the high blood pressure in this subset of patients remains to be elucidated, but is thought to be caused by chronic secondary hyperaldosteronism [30, 31].

Since the recycling of potassium across the luminal membrane creates the driving force for paracellular absorption of cations including calcium in the TAL, hypercalciuria causing nephrocalcinosis is typical for the “loop phenotype.” Nephrocalcinosis is also seen in autosomal dominant hypocalcaemia with Bartter-like syndrome, since hypercalciuria is a key symptom in this condition [3, 4]. In contrast, GS is characterized by the combination of hypermagnesuria with hypocalciuria. Hypermagnesuria is caused by decreased expression of the luminal magnesium channel TRPM6 in cells lining the DCT. The exact pathway leading to the downregulation of this transporter, however, remains unknown but is probably related to DCT cell dysfunction due to the mutations in NCC. The hypocalciuria that is typically seen in patients with GS is caused by increased passive reabsorption of calcium in the PT. This is an effect that is secondary to the extracellular volume contraction caused by increased sodium wasting. Volume depletion triggers an increase of sodium reabsorption in the PT, which in turn increases the electrochemical gradient that drives the passive calcium transport in this nephron segment [18, 19].

### Problems with the current classification

The initial classification of the different subtypes of BS mainly focused on the age at presentation, distinguishing antenatal variants of BS (including BS1, BS2, BS4, and BS5, all typically associated with severe polyhydramnios causing premature birth) from classical BS (BS3). This classification mainly serves the point of view of neonatologists who are faced with patients in their first weeks of life, having the complications of prematurity alongside the risk for severe fluid and electrolyte problems if not monitored and supplemented aggressively. However, some patients with BS3 can also present in the antenatal period with moderate polyhydramnios and premature birth [22]. Because their presentation is earlier than usually seen in classical BS, these patients are sometimes marked to have antenatal BS although their clinical phenotype is typically less severe compared to “true” antenatal BS, thus causing confusion when this classification is used.

Another way to classify BS and GS, which is focused on the underlying physiology, may be more appealing to the (paediatric) nephrologist’s point of view. When using this classification, BS is generally considered a disorder of TAL, whereas GS is a disorder of DCT function. Yet, confusingly, some subtypes of BS also affect the DCT and, in fact, BS3 can be clinically indistinguishable from GS [3, 4, 21, 28, 32, 33]. For this reason, a classification has been proposed, separating the various types into disorders of the TAL, the DCT, or of both segments [13]. Disorders of the TAL are characterized by hypercalciuria and nephrocalcinosis and are typically associated with an antenatal onset. Pharmacologically, they are equivalent to the use of loop diuretics. The genetic causes are usually mutations in *SLC12A1* (NKCC2) or *KCNJ1* (KCNJ1). In contrast, disorders of the DCT are characterized by hypo- or normocalciuria, hypomagnesaemia and typically manifest later in childhood or adolescence [3, 4, 21, 28]. The pharmacologic equivalent here is thiazide diuretics and the genetic causes are usually mutations in *CLCNKB* or *SLC12A3* (NCC). The “compound disorders” affecting both segments are usually caused by *BSND* (Barttin) or combined *CLCNKB/A* mutations and typically have a severe clinical phenotype with antenatal onset. Calcium excretion can be variable.

This physiology-based classification makes perfect sense when assessing patients at a given moment in time: if the phenotype mimics a loop or thiazide diuretic or the combination of the two, the patient can be classified as having a TAL, DCT, or combined disorder. But problems arise, when following patients over time. And again, the confusing phenotypes are those associated with mutations in CLCNKB. In fact, it is interesting that the authors of this physiology-based classification did not assign CLCNKB-based phenotypes to the “compound disorders,” although this channel, as discussed above, is expressed in both segments, TAL and DCT. Yet, clinically this makes sense, as most patients with BS type 4 (a and b) can easily be distinguished from BS3, based on their earlier antenatal presentation, more severe polyhydramnios and more complicated postnatal course (as well as the deafness) [34, 35]. Interestingly, BS3 can present either with a “loop” or a “DCT” phenotype, but not as a combined disorder [32]. In fact, some patients appear to “switch” phenotype, initially presenting with a “loop” or BS phenotype and, during later childhood and adolescence, develop the typical DCT or GS phenotype [36]. If one were to apply the physiology-based classification, then these patients would change diagnosis from BS to GS over time, creating enormous potential for confusion. There are also some clinical findings that argue against equivocating BS3 with GS: the age of onset is usually younger and complications, such as heavy proteinuria with focal segmental glomerulosclerosis on histology, which presumably reflects persistent hyperreninism, but also growth failure are virtually exclusively reported in BS3 [21, 32, 37–39]. For this reason, we propose to stick to a gene-based classification, especially in countries where genetic analysis is easily available.

Yet, apart from these problems with classification, the phenotypic “switch” raises fascinating questions over the underlying pathophysiology: is there another basolateral chloride exit pathway in the DCT that can provide some compensation for loss of CLCNKB function, so that initially the “loop” phenotype predominates? If so, why is this compensatory pathway apparently lost over time, so that the “DCT” phenotype subsequently emerges? Or, considering that GS typically presents in later childhood and adolescence, i.e., the time frame of the phenotypic “switch,” perhaps the initial “loop” phenotype just dominates the clinical picture in the first years of life? Yet, how come the “loop” phenotype disappears? Is the expression of CLCNKA increased over time, enabling it to compensate for the loss of CLCNKB in the TAL?

Unfortunately, these questions are difficult to address, as such a phenotypic “switch” has not been described in the Clc-k2 (the murine orthologue of CLCNKB) knock-out mice or any other animal model [5, 40]. Yet, clinical observations in other salt-wasting disorders have shown a similar development with time of the DCT phenotype, e.g., in EAST/SeSAME syndrome and the HNF1B-related tubulopathy [41, 42]. Further observations, coupled with investigations in the occasionally available biopsy tissue, or of “liquid” biopsies, e.g., urinary exosomes, may provide further insights over time.

### New insights

#### Bartter syndrome type 5

BS5 is the most recently discovered form of BS, and it raises some fascinating questions regarding the developmental regulation of salt reabsorption [24]. BS5 is inherited in an X-linked recessive manner. Male (and occasionally female) patients present antenatally with severe polyhydramnios, causing premature birth, sometimes before viability. But, most fascinatingly, this newly described type of BS is transient and symptoms resolve after the first weeks of life.

Genetic studies showed that this new BS subtype is caused by mutations in the gene encoding the protein MAGE-D2. Subsequent expression analysis revealed that MAGE-D2 is expressed in the TAL and more distal nephron segments in both developing and adult kidneys. It was shown that MAGE-D2 increases the expression of NKCC2 in the TAL and NCC in the DCT; in the absence of MAGE-D2, both transporters were trapped in the endoplasmic reticulum, resulting in decreased expression at the luminal cell membrane. The reason why this results in a transient phenotype remains to be elucidated. It was hypothesized that other proteins could influence NKCC2 and NCC expression during different stages of gestation. Also, MAGE-D2 function could be altered by the reduced tissue oxygenation due to reduced kidney perfusion that occurs physiologically after birth [24]. Meanwhile, more cases of this subtype of BS have been reported. In a large French cohort, 17 out of 42 unsolved cases of antenatal BS were found to be caused by mutations in the gene encoding MAGE-D2. In all babies that survived the neonatal period, BS was found to be transient. Interestingly, 2 of the reported patients were female: the severe phenotype could be explained by extremely skewed X-inactivation in 1 of them [43].

#### Urinary acidification and alkalinisation

Recently, new insights were gained in the urinary acidifying properties of the TAL. It was generally believed that urinary acidification after administration of loop diuretics, which block NKCC2 and therefore mimic BS, was caused by the increased load of sodium reaching the CD. In this nephron segment, sodium is reabsorbed by the principal cells via the specific sodium channel ENaC. The reabsorption of positively charged sodium results in a voltage gradient, which facilitates proton secretion by the type a intercalated cells via V-type H-ATPase. Continuous increased urinary acid secretion will finally cause metabolic alkalosis, as is seen in BS. Yet, there are two other potential pathways that could contribute:

Recent animal studies showed, however, that there is another urinary acidification system that is activated after the administration of furosemide. In the TAL, Nkcc2 co-localizes with the Na/H exchanger type 3 (Nhe3). After the administration of furosemide, luminal sodium reabsorption through Nkcc2 stops but sodium continues to exit the cell via the basolateral Na-K-ATPase. This will result in intracellular sodium depletion, which is the driving force for Nhe3. The latter reabsorbs sodium from the luminal fluid in exchange for protons, thus causing urinary acidification and intracellular alkalosis [44].

Moreover, it has been shown that the chloride transporter CLCNKB is also expressed on the basolateral side of type a (acid secreting) and type b (alkaline secreting) intercalated cells in the collecting duct. Although acid secretion by type a intercalated cells might be impaired in BS3, BS4a, and BS4b, this is counteracted by a loss of bicarbonate secretion by type b intercalated cells. The presence of an alkaline pH activates CLCNKB (thereby increasing bicarbonate secretion by type b intercalated cells), whereas the opposite is true in the presence of an acidic pH. Due to this specific pH response, impaired CLCNKB function in the collecting duct will mainly affect type b intercalated cells. This could explain the profound metabolic alkalosis that is typically seen in BS3 and BS4, which is more severe compared to other subtypes of BS [45, 46].

#### Paracellular sodium absorption in the TAL

In the healthy TAL, it is estimated that 50% of sodium is reabsorbed via the previously described transcellular route [47]. The other half is reabsorbed paracellularly via claudins in the tight junctions, which depend on the lumen-positive transepithelial potential. The tight junctions in the TAL either consist of claudin 10b or a complex of claudin 3, claudin 16, and claudin 19. Claudin 10b preferentially transports sodium over calcium or magnesium, whereas the complex of claudins 3, 16, and 19 preferentially transports divalent cations like calcium and magnesium [48].

Vasopressin can increase sodium reabsorption via the transcellular route by increasing NKCC2 expression and activity [49]. Increased sodium reabsorption in the TAL increases the tonicity of the interstitial space, which in turn favors the reabsorption of water in the CD. More recently, it was discovered that vasopressin can also increase sodium uptake via the paracellular route, possibly via claudin 10b [50]. Whether these recent findings on paracellular sodium transport could be beneficial in the search for possible treatment options for BS remains to be elucidated.

### Future considerations

Although most patients with BS and GS nowadays have a genetic confirmation of their diagnosis, there is still a significant group of patients with a BS phenotype in whom no disease-causing mutation could be detected. New techniques, including whole-exome/genome sequencing, could identify other genes that can cause BS and GS and increase our knowledge of the exact mechanisms of tubular solute handling [3, 4, 21]. For instance, the recent discovery that vesicle-associated membrane protein 3 (VAMP3) is needed for accurate intracellular trafficking of NKCC2 to the luminal membrane, and that *Vamp3*^*-/-*^ mice exhibit a BS phenotype, would make this an interesting candidate gene [51]. Also, mutations in genes involved in the phosphorylation of NKCC2 could decrease its activity. Adenylyl cyclase 6 (AC6) is a crucial step in the vasopressin-mediated phosphorylation of NKCC2. It has been shown in a mouse model that deficiency of Ac6 results in low Nkcc2 expression and a mild BS phenotype [49], which again would make this an interesting candidate gene in unsolved cases of BS. But it may not necessarily be new disease genes: for instance, we described a patient with a clinical diagnosis of GS, who subsequently was found to have a mutation in HNF1B [33].

Our knowledge of tubular transporters and factors influencing their expression is rapidly increasing, but an effect of this knowledge on the treatment of patients with tubulopathies still lags behind. The treatment of BS and GS is still purely symptomatic. In BS, the mainstay of treatment consists of adequate fluid intake, replacement of sodium and potassium, and minimizing urinary losses water and electrolytes by the administration of nonsteroidal anti-inflammatory drugs. Patients with GS are often treated with a high-salt diet, combined with potassium and magnesium supplements [3, 4, 21, 28]. Recently, an animal study was published in which gene therapy was used to successfully target the Nkcc2 gene with an adenoviral vector to the TAL *in vivo*. To prevent adenoviral uptake by the liver, the vector was directly injected into the renal artery. Specific promotors were designed to target the vectors to their assigned nephron segment. After transfection, exogenous and endogenous Nkcc2 were indeed co-expressed in the TAL [52]. This could be a first step towards gene therapy as a possible curative treatment for tubulopathies like BS and GS, although much more research is needed to ensure the long-term safety of the viral vectors used and to ensure the permanent transfection of the target gene in a specific cell type with the end result of a functioning transporter. Also, some recurrent mutations in the gene encoding KCNJ1 alter protein folding, which in turn results in these proteins being trapped in the endoplasmatic reticulum and targeted for destruction by the endoplasmatic reticulum-associated degradation pathway. These folding defects could be corrected at low temperature in yeast cells [53]. That is a promising finding, since in other diseases like cystic fibrosis folding defects that are correctable at low temperature in yeast cells can now be corrected in man using chaperone proteins [54]. Therefore, the possibilities to correct these KCNJ1 folding defects and restore protein function should be further studied and may provide a new therapy for a specific group of BS patients.

## Conclusion

Our knowledge regarding the transporters that are expressed in the TAL and the DCT is rapidly increasing, so is our knowledge about the exact mechanisms that cause a BS or GS phenotype. However, there is still much to learn about proteins that are involved in the expression and activity of these transporters. Since there is still a subgroup of patients in whom no mutation can be found in the genes that are currently known to cause BS, the investigation of genes involved in gene expression, protein trafficking, or transporter activity could result in the discovery of new subtypes of the disease. Moreover, the current treatment of BS and GS is only symptomatic. Future studies should focus on possible curative treatments for these diseases. The recent discoveries on how vasopressin can increase paracellular sodium transport in the TAL as well as studies performed using gene therapy to successfully express NKCC2 in a murine model could be of importance to find possible treatments for these conditions.
